# Supplementary material for: tRNA m1G9 modification depends on substrate-specific RNA conformational changes induced by the methyltransferase Trm10
Source: J Biol Chem. 2023 Nov 8;299(12):105443. doi: 10.1016/j.jbc.2023.105443 (PMC10704376; doi:10.1016/j.jbc.2023.105443)
Supplement: Supplemental Figs. S1–S8 and Table S1 [file mmc1.pdf]

SUPPORTING INFORMATION

tRNA m^{1}G9 modification depends on substrate-specific RNA conformational changes induced by the methyltransferase Trm10

Sarah E. Strassler[1,2], Isobel E. Bowles[3,4], Aiswarya Krishnamohan[3,4], Hyejeong Kim[3], Catherine B. Edgington[3,4], Emily G. Kuiper[1,2], Clio J. Hancock[1], Lindsay R. Comstock[5], Jane E. Jackman[3*] and Graeme L. Conn[1,2*]

This file contains:

- Supplementary Figures S1-S8
- Supplementary Table S1

SUPPLEMENTAL FIGURES

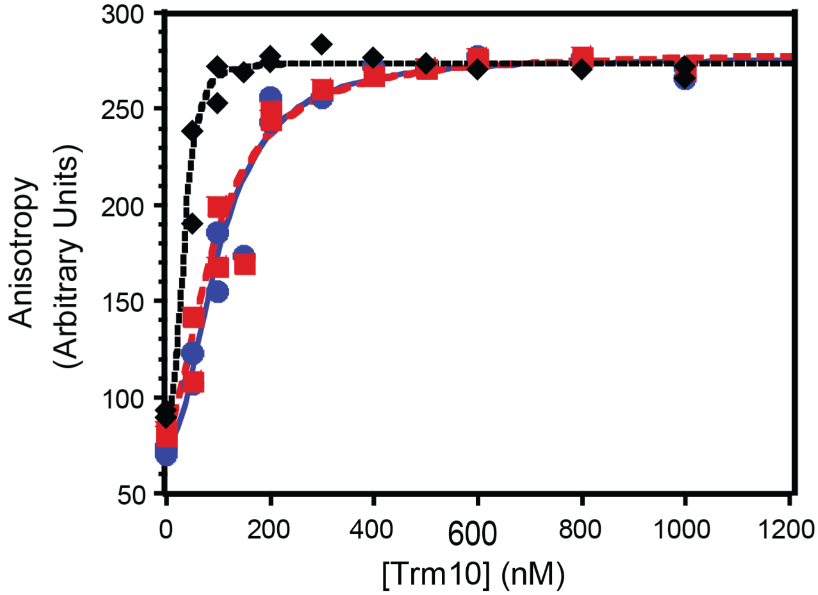

Supplemental Figure S1. Binding affinity of tRNAs with Trm10. Wild-type Trm10 binding affinity was determined by fluorescence anisotropy for $tRNA^{Gly\text{-}GCC}$ (*blue circles*; K_D = 100 ± 8 nM), $tRNA^{Val\text{-}UAC}$ (*red squares*; K_D = 96 ± 10 nM), and $tRNA^{Leu\text{-}CAA}$ (*black diamonds*; K_D = 38 ± 5 nM). Data from three independent experiments were plotted together and fit to equation 1 (see *Experimental Procedures*).

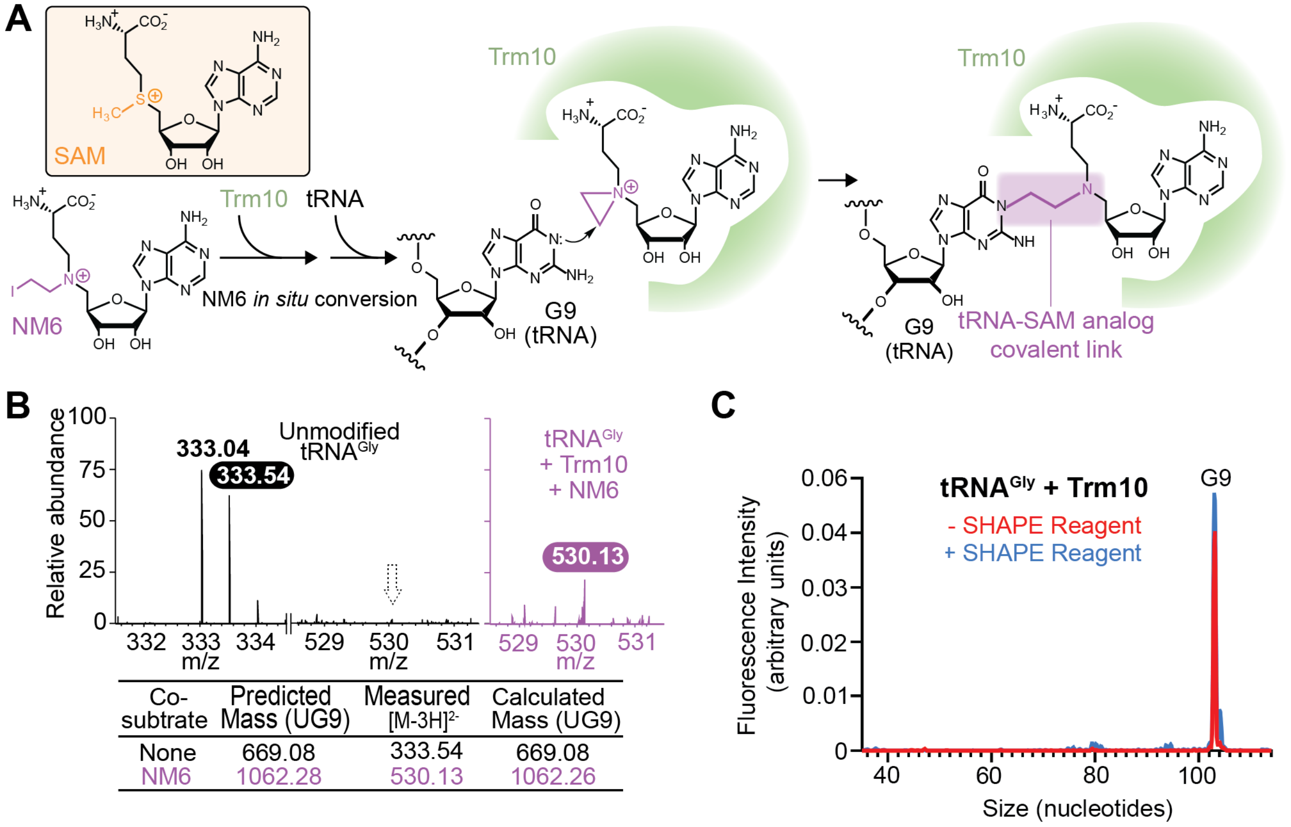

Co-subtrate	Predicted Mass (UG9)	Measured $[M-3H]^{2-}$	Calculated Mass (UG9)
None	669.08	333.54	669.08
NM6	1062.28	530.13	1062.26

Supplemental Figure S2. Stabilization of the Trm10-tRNA complex using the SAM analog NM6. *A*, N-mustard 6 (NM6; *bottom left*) is an analog of S-adenosyl-L-methionine (SAM; *top left box*) that Trm10 can use as a cosubstrate for modification of tRNA. In this enzymatic reaction, NM6 becomes covalently attached to the tRNA at the N1 base position of G9. The Trm10-tRNA complex is thus stabilized in a state immediately following catalysis by virtue of Trm10's affinity for both tRNA subunit and the cosubstrate analog covalently attached to G9. ***B***, The covalent attachment of NM6 to G9 of $tRNA^{Gly}$ is confirmed by MS analysis showing a peak at an m/z of 530.13 representing the UG_9-NM6 fragment. This peak is not observed in the sample in which NM6 is absent (dotted arrow). ***C***, Example capillary electrophoresis chromatogram of $tRNA^{Gly\text{-}GCC}$ in the presence of Trm10 and NM6 displaying a large peak at the modification site, indicating that the attachment of NM6 prevents further reverse transcription.

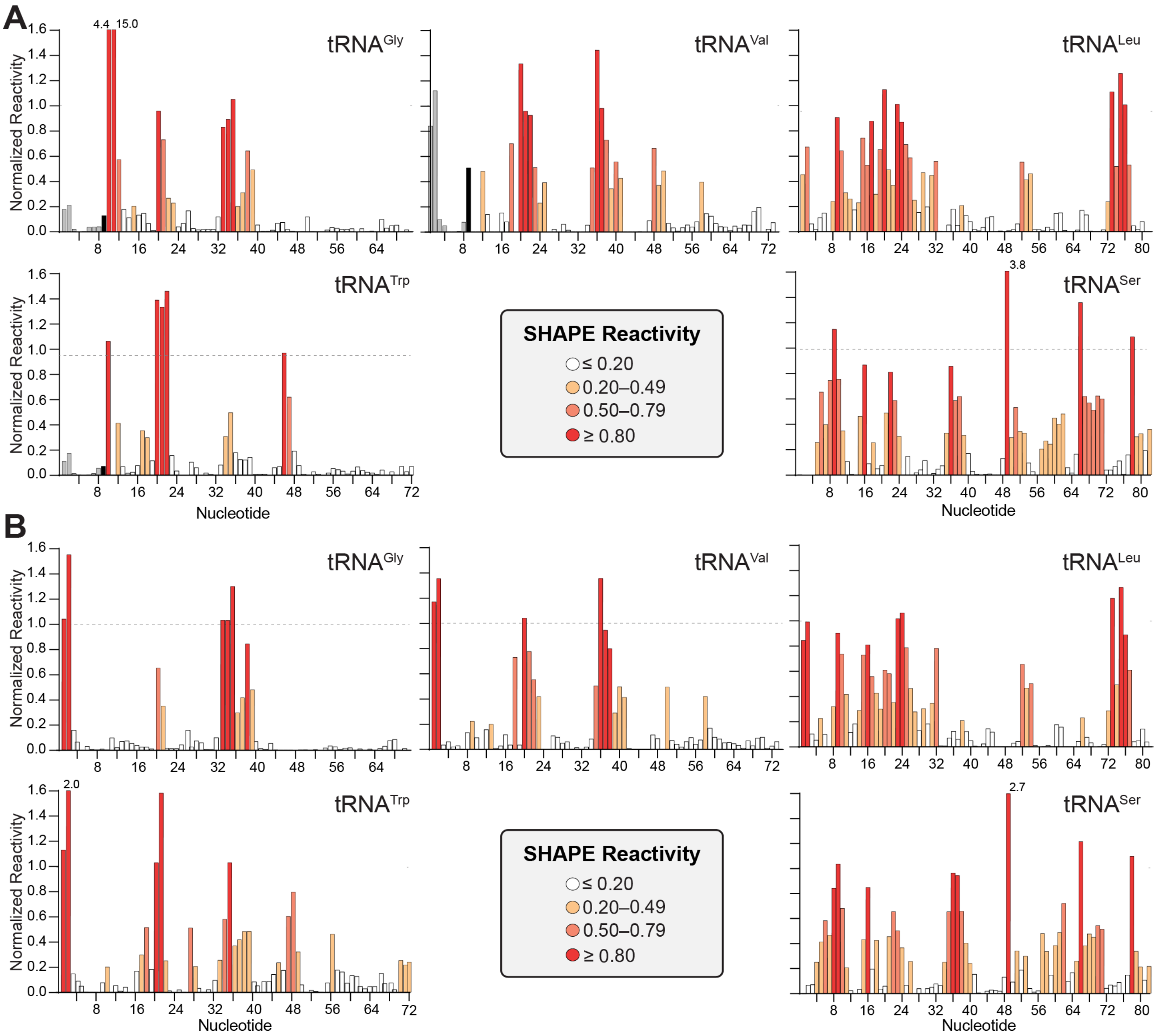

Supplemental Figure S3. Normalized reactivities of tRNAs bound to wild-type Trm10 and Trm10-KRR. SHAPE reactivities of tRNA bound to ***A***, wild-type Trm10 or ***B***, Trm10-KRR were normalized by dividing each value by the average of the reactivities of the highest 8%, omitting the highest 2%. The values from two replicates were then averaged and classified as shown in the key in the center of the bottom row of each panel.

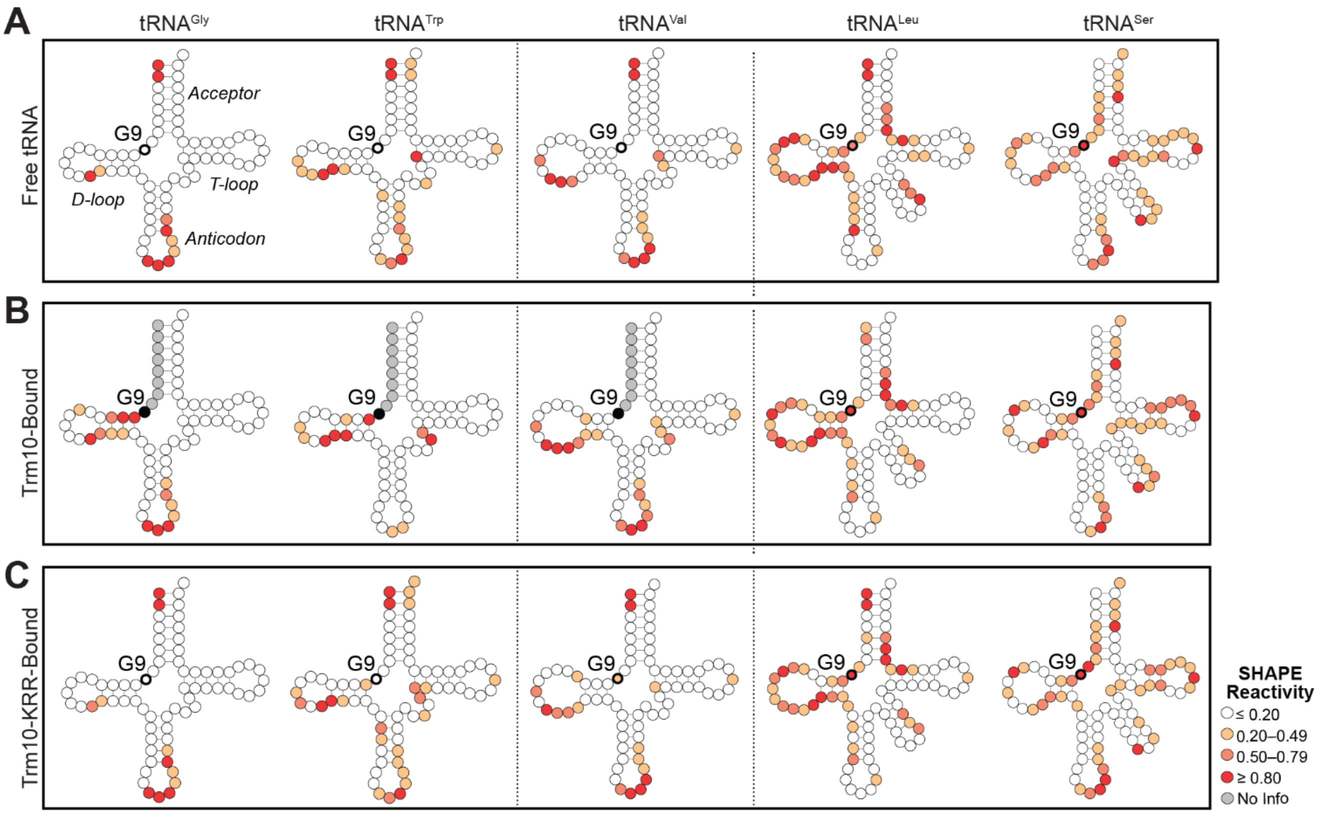

Supplemental Figure S4. Nucleotide SHAPE reactivities in free and Trm10-bound tRNAs. SHAPE reactivities mapped onto tRNA secondary structures for: ***A***, free tRNA (note, these data are the same as shown in **Fig. 2B**), ***B***, tRNA bound to wild-type Trm10, and ***C***, tRNA bound to Trm10-KRR. The color scale for SHAPE reactivity is shown in the *bottom right.*

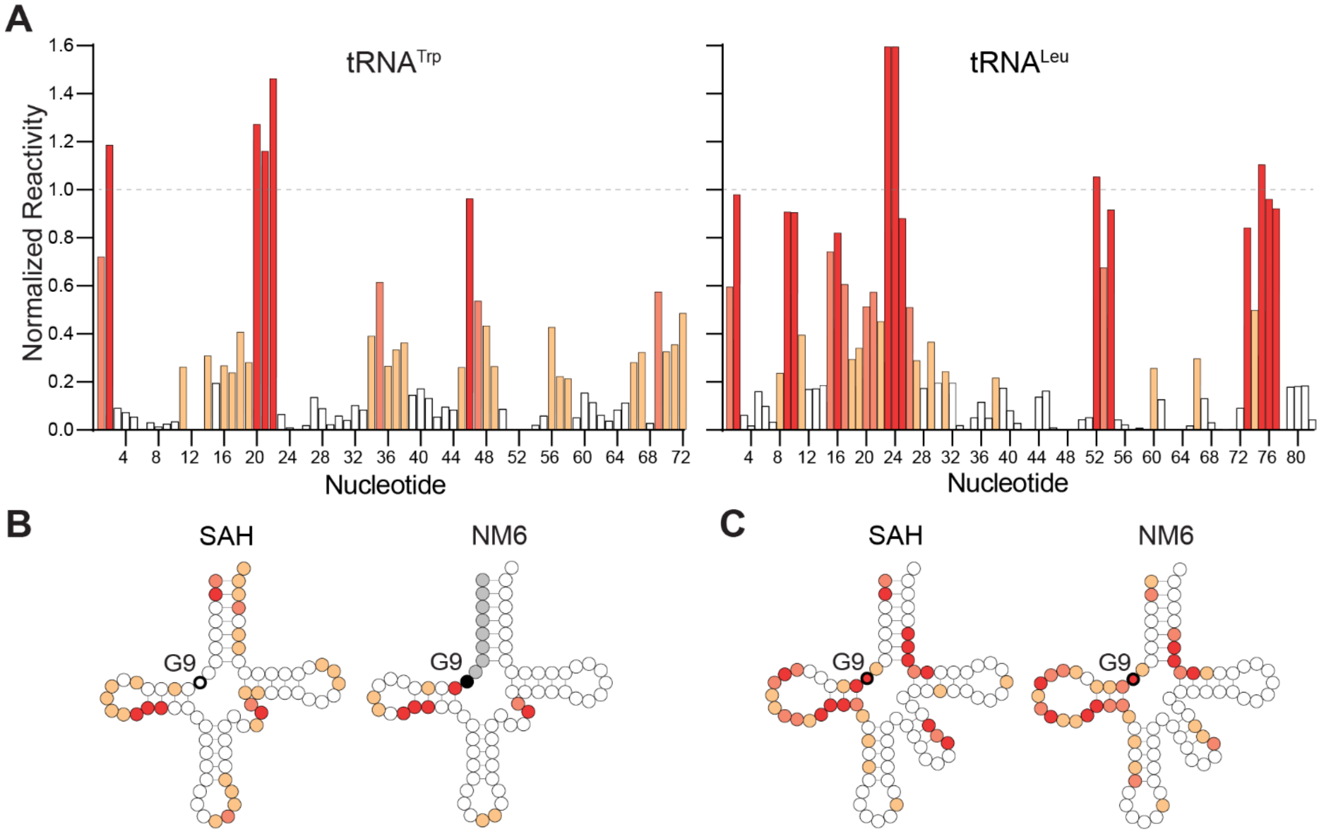

Supplemental Figure S5. SHAPE reactivities of substrate tRNA^{Trp} and nonsubstrate tRNA^{Leu} bound to wild-type Trm10 in the presence of SAH. ***A***, SHAPE reactivities of tRNA^{Trp} (substrate) and tRNA^{Leu} (nonsubstrate) bound to wild-type Trm10 in the presence of SAH were normalized and the values for two replicates were averaged. SHAPE reactivities of ***B***, tRNA^{Trp} and ***C***, tRNA^{Leu} bound to wild-type Trm10 in the presence of cosubstrate SAH or NM6 (note, these data for NM6 are the same as shown in **Supplemental Figure S4B**) are mapped onto the tRNA secondary structure. The color scale for SHAPE reactivity is shown for both panels in the *bottom right.*

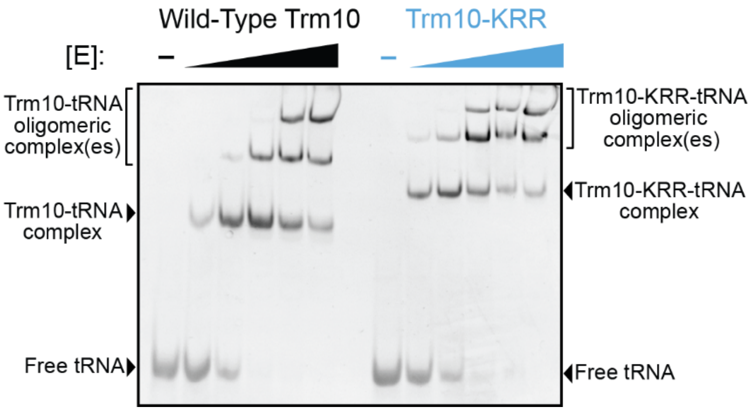

Supplemental Figure S6. Electromobility shift assay (EMSA) of substrate tRNA^{Gly} with wild-type Trm10 and Trm10-KRR. tRNA^{Gly} was incubated with increasing concentrations of wild-type Trm10 or Trm10-KRR and samples were run on a non-denaturing 10% polyacrylamide gel. The apparent differences in mobility of the mutant complex are likely due to the different electrostatics of the enzyme-tRNA complex(es) but overall similar binding affinities and patterns of complex formation are observed between wild-type Trm10 and Trm10-KRR.

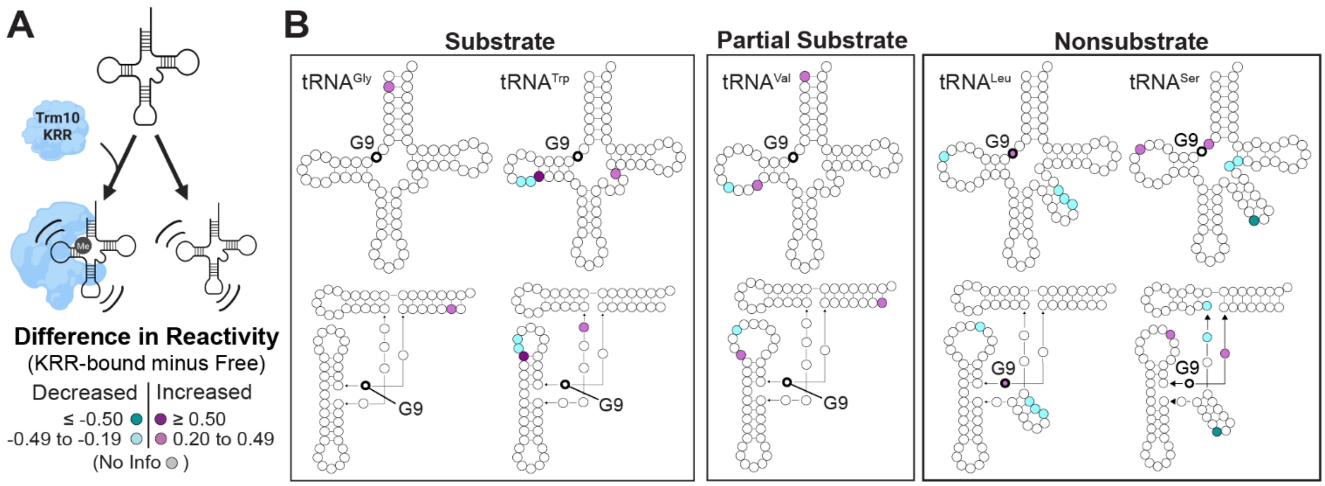

Supplemental Figure S7. Comparison of unbound tRNA and KRR-bound tRNA. ***A***, Schematic of comparison being made between free tRNA and KRR-bound tRNA. ***B***, Difference SHAPE reactivities between KRR-bound and free tRNAs mapped onto secondary (*top*) and tertiary (*bottom*) structures for (*left* to *right*): $tRNA^{Gly\text{-}GCC}$ (substrate), $tRNA^{Trp\text{-}CCA}$ (substrate), $tRNA^{Val\text{-}UAC}$ (partial substrate), $tRNA^{Leu\text{-}CAA}$ (nonsubstrate), and $tRNA^{Ser\text{-}UGA}$ (nonsubstrate). The color scale for difference in SHAPE reactivity (KRR-bound minus free tRNA) is shown in the *bottom left*.

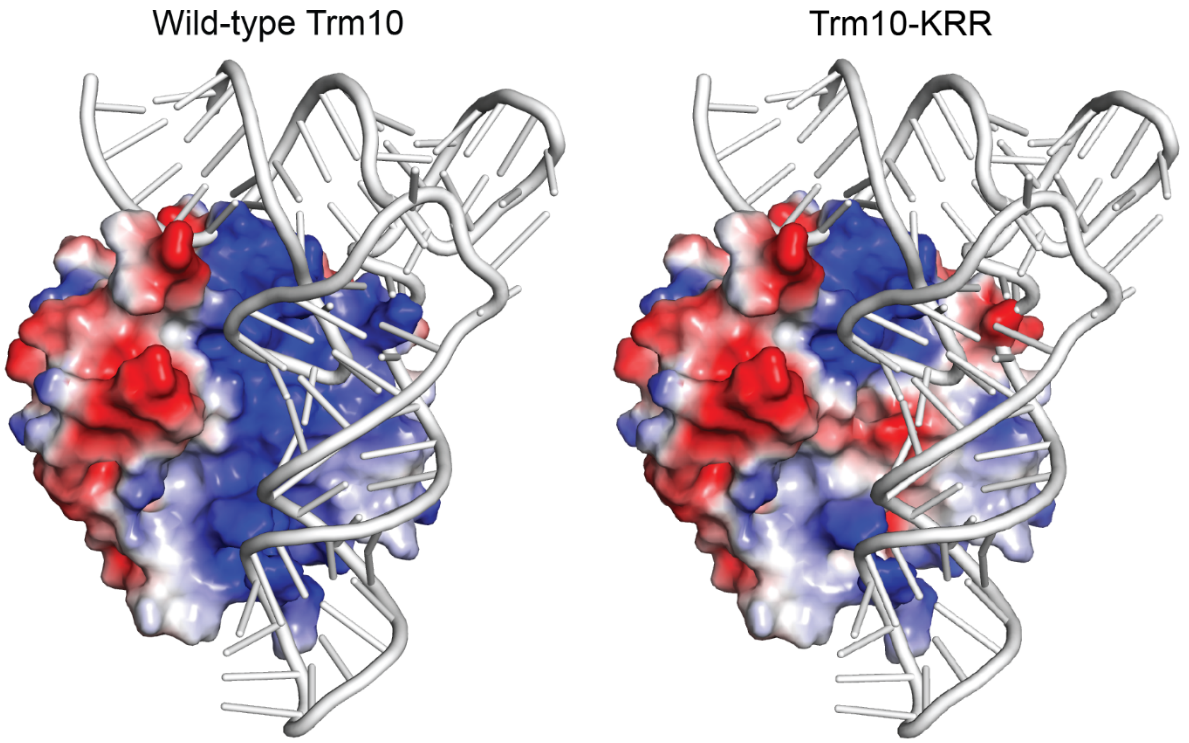

Supplemental Figure S8. Electrostatic surface potential of wild-type Trm10 and Trm10-KRR shown on a Trm10-tRNA model. Amino acid substitutions to *S. cerevisiae* Trm10 C-terminal domain (PDB 4JWJ) were made using the PyMol mutagenesis tool to generate the Trm10-KRR variant. Protein electrostatics were then generated for wild-type Trm10 and Trm10-KRR in PyMol and are shown on a semi-transparent surface representation for each protein. The placement of tRNA was predicted from the structure of TRMT10C in complex with pre-tRNA (PDB: 7ONU) and is shown using $tRNA^{Phe}$ (PDB: 6LVR).

SUPPLEMENTAL TABLES

Supplemental Table 1. Association constants (K_A) for Trm10 binding SAM and SAH determined by ITC.

		SAM[a]	SAH[a]
K_A (M^{-1})	Wild-Type Trm10	$8.90 \pm 0.27 \times 10^4$<br>$11.9 \pm 0.87 \times 10^4$	$5.1 \pm 1.42 \times 10^4$<br>$4.62 \pm 1.33 \times 10^4$
	Trm10-KRR	$32.5 \pm 1.83 \times 10^4$<br>$33.1 \pm 0.88 \times 10^4$	$10.7 \pm 1.92 \times 10^4$<br>$7.96 \pm 0.70 \times 10^4$

[a]Replicate measurements shown with the direct fit error determined using a model for one-binding site in Origin software after subtraction of residual heats.
